# Response of *Coccomyxa cimbrica* sp.nov. to Increasing Doses of Cu(II) as a Function of Time: Comparison between Exposure in a Microfluidic Device or with Standard Protocols

**DOI:** 10.3390/bios13040417

**Published:** 2023-03-23

**Authors:** Riccardo Speghini, Carlo Buscato, Stefania Marcato, Ilaria Fortunati, Barbara Baldan, Camilla Ferrante

**Affiliations:** 1 Dipartimento di Scienze Chimiche, Università degli Studi di Padova, 35131 Padova, Italy; riccardo.speghini@gmail.com (R.S.); c.buscato7@gmail.com (C.B.); ilaria.fortunati@unipd.it (I.F.); 2 Dipartimento di Biologia, Università degli Studi di Padova, 35131 Padova, Italy; stefania.marcato@unipd.it (S.M.); barbara.baldan@unipd.it (B.B.)

**Keywords:** microfluidic, microalgae, fluorescence imaging, FLIM, Cu(II) toxicity

## Abstract

In this study, we explore how the in vitro conditions chosen to cultivate and observe the long-term (up to 72 h) toxic effect of Cu(II) on the freshwater microalga *Coccomyxa cimbrica* sp.nov. can affect the dose response in time. We test three different cultivation protocols: (i) under static conditions in sealed glass cells, (ii) in a microfluidic device, where the sample is constantly circulated with a peristaltic pump, and (iii) under continuous agitation in plastic falcons on an orbital shaker. The advantage and novelty of this study resides in the fact that each condition can mimic different environmental conditions that alga cells can find in nature. The effect of increasing dose of Cu(II) as a function of time (24, 48, and 72 h) is monitored following chlorophyll *a* fluorescence intensity from single cells. Fluorescence lifetime imaging experiments are also explored to gain information on the changes induced by Cu(II) in the photosynthetic cycle of this microalga.

## 1. Introduction

Microalgae are unicellular organisms which can exist as individuals, in chains, or in groups. They are present in marine and freshwater environments. These organisms are of enormous importance, as they contribute massively to oxygen production through the photosynthetic cycle. At the same time, they have been investigated for the capability to produce lipids and, therefore biofuels [1] and for the removal of contaminants in wastewaters, given the high tolerance shown for many inorganic and organic toxicants [2].

Among toxicants, the metal ion Cu(II) has a strong effect on the growth and viability of aquatic microorganisms, in particular, microalgae. Cu(II) is always present in culture media as well as in the natural environment because it is an essential micronutrient. However, high doses of this metal induce adverse effects, such as ROS (Reactive Oxygen Species) production, which, in turn, affects many metabolic pathways and the photosynthetic efficiency of the microalgae. Many studies have been devoted to this last topic, and the observation of changes in the spontaneous fluorescence of chlorophylls in the photosystems allows evaluation of the insurgence of stress in the cell [3]. In particular, changes in the fluorescence intensity following pulsed excitation (PAM: pulse amplitude modulated fluorescence), allow exploration of the way in which the photosynthetic cycle is affected by Cu(II) [4,5,6,7]. Other fluorescence techniques used to evaluate Cu(II) toxicity are flow cytometry [8,9] and fluorescence microscopy using both autofluorescence and staining with fluorescent labels.

Since its inception in the late 1990s, fluorescence lifetime imaging microscopy (FLIM) has become a valuable technique to monitor the changes in decay dynamic of dyes labeling biological constructs. It has been extensively used to characterize structural changes and biochemical signaling in in vitro and in vivo experiments on many different types of cells [10,11,12] In plant cells and microorganisms, FLIM experiments have supplied information on the dynamic of the photosynthetic process [13,14]. Few FLIM experiments have also been devoted to the characterization of toxicants activity in microalgae [15,16]

Almost all the studies assessing microalgae are based on in vitro experiments in which microalgae are cultivated in flasks or in microwells. In the last decade, microfluidic devices have shown to be an interesting alternative for the growth, manipulation, and characterization of cell cultures. Furthermore, the realization of microfluidic devices can be easily accomplished without the need for specialized fabrication facilities and research equipment [17].

Microfluidic devices have been extensively used for the investigation of animal and human cells [18,19,20]. Some of the key features characterizing these devices include a limited amount of supplies needed to maintain cell cultures, possibility to generate an environment capable to reproduce the natural conditions, and realization of co-cultures that mimic real organs.

Until now, extension of such devices to plant cells has been limited, although there is a growing trend, especially in the field of microalgae [21,22]. Prototype “lab on a chip” devices have been developed for the identification and selection of microalgal species characterized by a high level of lipids, useful in the field of biofuel production [23,24,25]. Among these, a prototype microfluidic chip for the cultivation and lipid extraction in aqueous isopropanol displayed a higher efficiency compared with conventional bulk methods [26].

Microfluidic chips, developed for microalgae cultivation, endorsed different growth strategies: continuous flow or droplet formation. Continuous flow microreactors are formed by culture compartments in which alga cells are confined in volumes from nano- to micro-liter scales, and the growth medium flows through small gaps that allow fluid exchange but block cell motion [27]. In droplet formation microfluidic reactors, few algae cells are encapsulated in aqueous droplets of few picoliters dispersed in an oil medium. The droplets can be collected in larger chambers, where cultivation occurs under stationary conditions [28], or flown in single file along suitable microfluidic channels for inspection and manipulation [29].

Microfluidic chips have also been exploited to assess pollutant toxicity in the marine environment through observation of the motility change of phytoplankton exposed to heavy metal ions (Cu, Pb, Hg) or phenol. One of these chips was formed by four different units, generating a concentration gradient, connected to downstream diffusion chambers in which the phytoplankton is confined [30] The concentration gradient generator allows testing of eight different concentration of toxic agents on the same chip. Drastic changes in phytoplankton motility was observed after 2 h, and the EC_50_ values for motility inhibition of *P. subcordiformis* and *P. helgolanidica var. tsingtaoensis* fell approximately 2.4 and 5.3 μmol L^−1^ for Cu(II), respectively. Another microfluidic device [31] was based on a digital microfluidic diluter chip forming single droplets containing the microalgae and polluted seawater with different concentration gradients. Four different microalgae species were addressed, and their motility recorded with a camera after 2h exposure to the toxicants. The toxic agents used were Pb, Cu, phenol, and nonylphenol. The EC_50_ values for motility inhibition for *P. subcordiformis* are in the μmol L^−1^ range for both Pb and Cu. The major strength of these devices is the fast response time compared with classical protocols; on the other hand, they can be applied only on mobile microalgae endowed with a flagellum or cilia.

Other microfluidic devices rely on the autofluorescence of green microalgae to assess water pollution. A portable prototype chip, [32] integrating both the light source and the fluorescence detector, has been tested on *Chlamydomonas reinhardtii* using the herbicide Diuron as pollutant.

Long time exposure, up to 72 h, of microalgae to pollutants was achieved with a droplet-based microfluidic chip [33], where 500 nl droplets, containing the microalgal solution together with the pollutant, are continuously flown in a long tube coil. The toxicant effect on cell density was evaluated by means of transmission and fluorescence measurements. Experiments were carried out on *Chlorella vulgaris*, and CuCl_2_ was used as the toxic agent. The EC_50_ concentration, after 72 h exposure, fell between 34.6 and 39.9 μg mL^−1^. Experiments in microtiter plates showed a lower EC_50_ value of 7.8 μg mL^−1^, and the author attributed this change to an increase in alkalinity in microtiter plates caused by higher photosynthesis levels.

In this paper, we will describe three different protocols used to investigate the long-term toxic effect of Cu(II) on the freshwater microalga *Coccomyxa cimbrica* (*C. cimbrica*) [34] Among these, two are typical protocols used in toxicity experiments: small home-made sealed glass cells mimicking multiwells with no stirring of the solution and storage in large falcons under continuous shaking. The third one instead makes use of a microfluidic device that allows continuous flow of the algal solution together with the ability to isolate the sample from the environment as in sealed microwells. The effect of increasing doses of Cu(II) in time is tracked by confocal fluorescence imaging microscopy. In these experiments, the intensity of spontaneous chlorophyll emission from single cells is measured, and the statistical distribution of these single-cell emissions is analyzed. The advantage and novelty of this approach resides in the fact that each growth condition can mimic different environmental conditions that the alga cells can find in nature. In addition, FLIM was carried out to gain some insight in the change of the decay path of chlorophyll *a* for microalga *C. cimbrica* when exposed to increasing doses of Cu(II). Only the samples grown and exposed to Cu(II) in plastic falcons under continuous agitation were examined with FLIM.

## 2. Materials and Methods

Samples of microalga *C. cimbrica* were grown in Murashige and Skoog ½ medium with the addition of sucrose (2% *w*/*w*) and buffered at pH 5.5. All cultures were maintained at room temperature under a photoperiod of 16 h light/8 h darkness. CuCl_2_ from Merck was used to prepare cultures of *C. cimbrica* with different concentrations of Cu(II) in the range 10–1000 μg/mL. All experiments were carried out on fresh *C. cimbrica* samples in the exponential growth phase. Exposure to CuCl_2_ solution is carried out under different culturing conditions (hereafter, distinguished as Batches 1, 2, and 3): for Batch 1, we sealed the samples in home-made glass cells; in Batch 2, the solutions were continuously flown in a microfluidic device; and in Batch 3, samples were stored in 15 mL plastic falcons on an orbital shaker. The home-made cells of the first batch were formed by two glass microscope coverslips (0.150 mm thickness) separated by a double-sided sticky plastic gasket. The sample (0.5 mL) was stored in the glass cell for the entire time (72 h).

The microfluidic device for Batch 2 was formed by a linear channel engraved in silicon and attached on a rectangular coverslip glass slide [35]. The inlet and outlet at the ends of the channel were connected by sterile tubes to a sealed falcon tube, acting as reservoir, and to the inlet of a peristaltic pump, which continuously flowed 10 mL of solution in the device at a rate of 1 mL/min (a simple scheme and photographs are shown in Supplementary Materials Figure S1). The glass slide with the microfluidic chip was detached from the pump and reservoir when fluorescence measurements were carried out and then reattached. In Batch 3, 10 mL of cell cultures were sealed in plastic falcons, which were constantly stirred by an orbital shaker. For the fluorescence experiments, 0.5 mL of sample was collected each time and sealed in the same type of glass cells used for Batch 1 and afterwards discarded.

The effects of CuCl_2_ addition were followed by recording the spontaneous fluorescence of chlorophyll *a* present in the algae. A laser scanning confocal fluorescence microscope (Olympus Fluoview FV300, Evident Europe GmbH, Milan, Italy) allowed recording images of the fluorescence intensity as well as the FLIM maps for single cells. For the first and second batch, only single-cell fluorescence intensity images were recorded. Cells were excited by a CW Ar laser at 488 nm with average power on the sample of 4 μW. The laser was automatically scanned with both a 20× and a 60× microscope objective on a 700 × 700 μm and a 230 × 230 μm area, respectively. The fluorescence signal was selected by 565 nm longpass filter to reject laser light and recorded with a photomultiplier tube. For the third batch, instead, both fluorescence intensity images and FLIM maps were measured with the same confocal microscope but excited with a frequency doubled Ti-sapphire laser emitting 150 fs long pulses at 410 nm with a repetition rate of 76 MHz and an average power on the sample of 40 μW [36]. The fluorescence intensity pictures were recorded with the same set-up (filters and detector) used for Batch 1 and 2. For FLIM maps, instead, a 600 nm longpass filter was placed in front of a single photon avalanche photodiode (SPAD DPM from MPD, Bolzano, Italy) to record the signal. TCSPC fast electronic from Picoquant, Berlin, Germany, (PicoHarp 300) was used to record the fluorescence decay curves. FLIM maps were recorded for single cells on an area of 128 × 128 pixels, and each pixel had dimension of 100 nm.

FLIM measured the decay curves of the fluorescence intensity for each pixel of the scanned area. The time resolution of the single photon counting electronic was 100 ps. Fluorescence decay curves were recorded at each illuminated pixel of the microscope grid and were fitted with a multi-exponential function by the software SymPhoTime 64 (Picoquant):

$$I\left(t\right)=\sum_{i}A_{i}exp\left(-\frac{t}{\tau_{i}}\right)$$

where *I(t)* is the fluorescence intensity as a function of time, and *τ_i_* and *A_i_* are the characteristic decay time and the associated amplitude, respectively. An example of such a decay curve is given in Figure S2 of the Supplementary Materials. In our experiments, data are well fitted by three exponential components.

The first one is faster than the instrument time resolution and can have contributions from scattering of the incident laser light as well as very fast fluorescence decay. Because of this, the characteristic time of this component was fixed at 100 ps. The second and the third components fall in the ranges 200 ÷ 500 ps (*τ*_1_) and 1000 ÷ 2300 ps (*τ*_2_) and are both attributed to the fluorescence decay of chlorophyll *a* present in the cells.

Starting from these parameters, it is possible to generate different types of images of the cells. In this work, maps showing the intensity-weighted average decay time and the amplitudes associated with the two characteristic decay times are considered and discussed. FLIM maps for 10 different *C. cimbrica* cells were recorded for each one of the 5 different Cu(II) doses: 0, 30, 100, 300, 500, and 700 mg/mL and at each time interval: 0, 24, 48, and 72 h.

## 3. Results and Discussion

**Figure 1:**
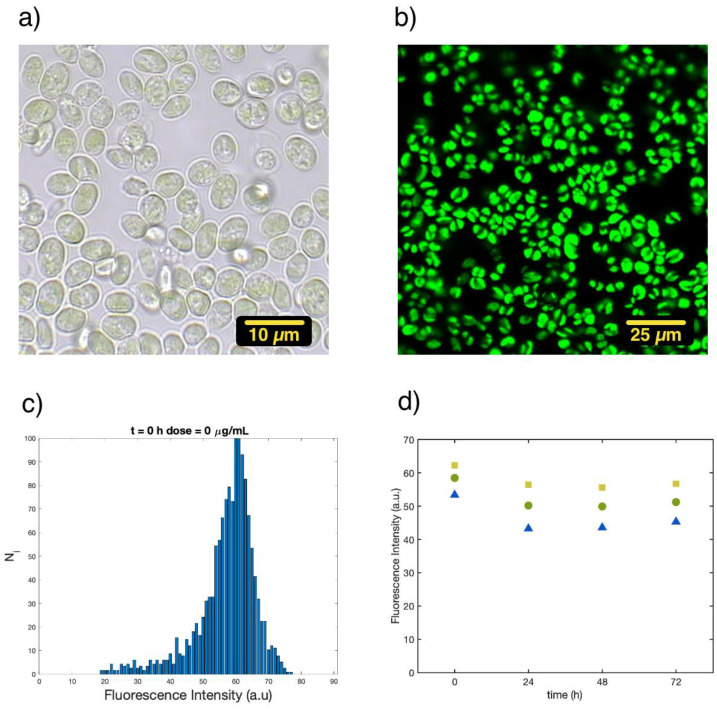
All pictures refer to a sample of *C. cimbrica* in buffer solution at room temperature: (**a**) transmission image recorded with white light (Batch 3), (**b**) spontaneous fluorescence emission excited at 410 nm (Batch 3), (**c**) histogram of the average fluorescence intensity of each single cell measured in pictures analogous to 1b (Batch 1), (**d**) 25th (blue triangles), 50th (green circles), and 75th (yellow squares) percentiles for the distribution of the average fluorescence intensity per single cell as a function of time (Batch 1).

Figure 1 summarizes the measurements of single cell fluorescence intensity and the way in which these data were elaborated. Figure 1a shows a typical image measured in the transmission mode with white light of *C. cimbrica* cells lying on the glass coverslip and immersed in the culture solution at room temperature. Figure 1b is just an image of the spontaneous fluorescence of the *C. cimbrica* cells from the same sample. At least four different pictures analogous to Figure 1b were taken for each sample so that the number of fluorescent cells measured was larger than 200. The average fluorescence intensity from each single cell was automatically evaluated with the software ImageJ. Figure 1c shows the histogram of this distribution: on the x-axis is the average fluorescence intensity of the cell in arbitrary unit, and the y-axis shows the normalized number of cells with such average intensity. Normalization is accomplished, assigning a value of 100 to the largest number of cells having the same intensity with the formula:

$$N_{i}=\frac{N_{i}^{S}\times 100}{N_{max}^{S}}$$

where $N_{i}^{S}$ and $N_{max}^{S}$ are the number of cells with average intensity *i* and *max*, respectively, and *N_i_* is the normalized value of cells having average intensity *i*. Histogram 1c reports the effect of Cu(II) on single cells, as in flow cytometry, providing the possibility to observe whether the cell population shows distinct distributions that can be attributed to “healthy”, “stressed”, or “dead” cells. Figure 1d shows the values of the 25th, 50th (median), and 75th percentiles of the distribution as a function of time: from 0 to 72 h. From the median, we can track the global change of the fluorescence intensity with dose and time, whereas the distance between the 25th and 75th percentiles is related to the change in width of the distribution.

### 3.1. Single-Cell Fluorescence Intensity Imaging

The effect of increasing doses of Cu(II) is monitored by looking at the spontaneous fluorescence arising from the emission of chlorophyll *a* molecules. Fluorescence is only one of the possible decay paths after excitation for chlorophyll *a*. Changes in fluorescence intensity or decay time can be related to stress condition as well as cell death. In our work, such stress and death are provided by the administration of increasing doses of Cu(II) to the growing medium.

**Figure 2:**
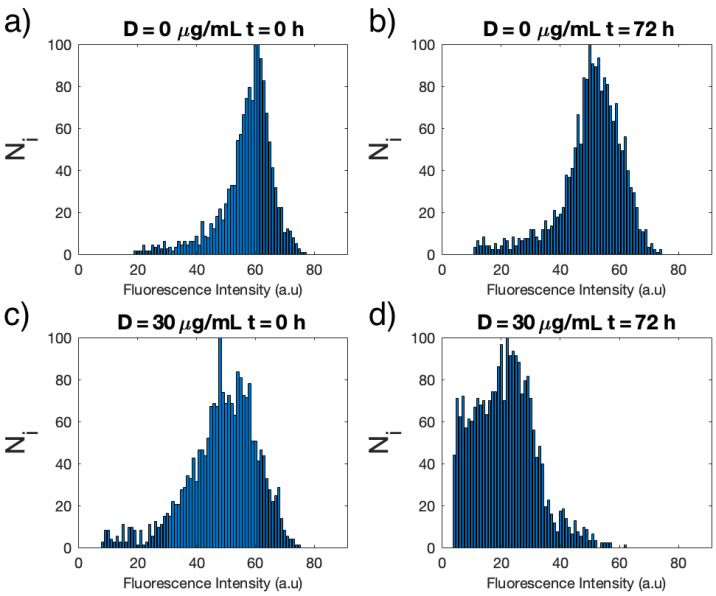
Histograms of the distribution of the average fluorescence intensity for each single cell of *C. cimbrica* of Batch 1 measured in: growth medium only (D = 0) at time t = 0 (**a**) and at t = 72 h (**b**) and with addition of 30 μg/mL (D = 30 μg/mL) of Cu(II) at t = 0 (**c**) and t = 72 h (**d**).

Batch 1:

The change in fluorescence intensity is monitored for each single cell and plotted in a histogram. Figure 2 shows the histograms at 0 and 72 h for *C. cimbrica* cells in the growth medium and with the addition of 30 μg/mL of Cu(II).

At t = 0, the average fluorescence intensity for the single cells without (Figure 2a) and with Cu(II) (Figure 2c) are similar, whereas the width of the intensity distribution is slightly narrower for the unexposed cells. After 72 h, the average intensity of the unexposed cells (Figure 2b) is slightly smaller than at time t = 0, but the distribution is still narrow. On the other hand, cells exposed to 30 μg/mL Cu(II) show a strong decrease in the average fluorescence intensity as well as an even larger distribution.

**Figure 3:**
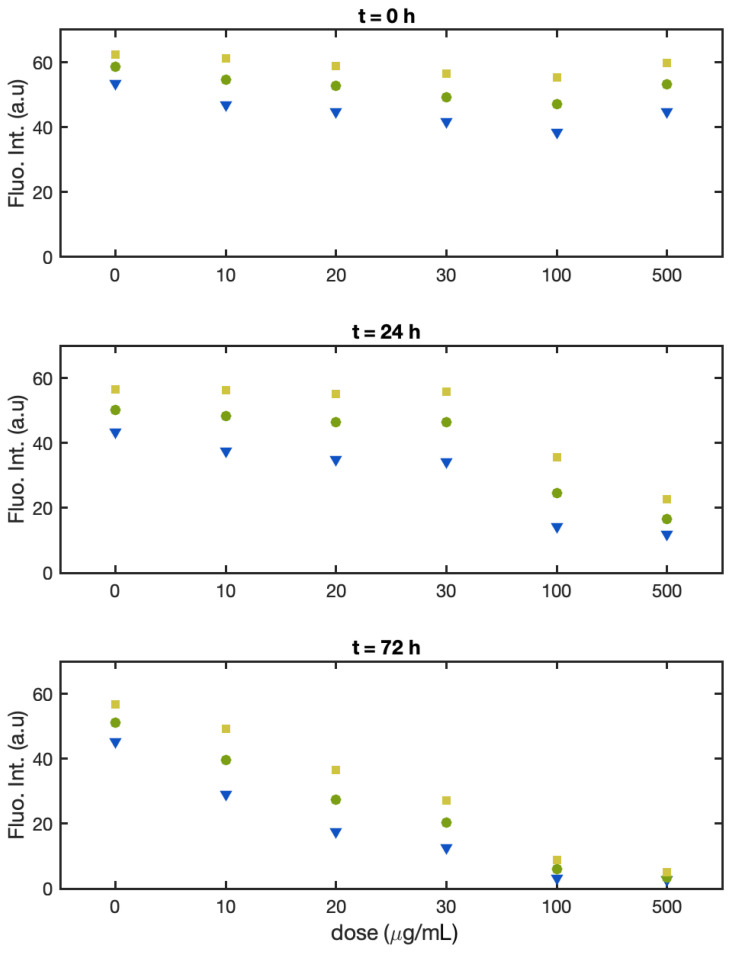
Batch 1: Normalized fluorescence intensity of the single alga cells at the 25th percentile (yellow square), median (green dot), and 75th percentile (blue triangle) as a function of Cu(II) concentration at t = 0 (**upper panel**), t = 24 h (**middle panel**), and t = 72 h (**lower panel**).

Figure 3 shows how the single-cell fluorescence intensity distribution for cells of Batch 1 evolves as a function of the Cu(II) dose for concentrations between 10 and 500 μg/mL and in time at 0, 24 h, and 72 h. The triangles, circles, and squares represent the 25th, median, and 75th percentiles of the single-cell fluorescence intensity. At time zero (Figure 3, upper panel), the fluorescence intensity falls in the same range for all solutions, and only the width of the distribution is slightly larger for cells exposed to Cu(II). After 24 h (Figure 3, middle panel), all samples, including the control (no Cu(II)), show a 10–15% decrease in fluorescence intensity up to a concentration of 30 μg/mL. Only at 100 and 500 μg/mL is there a marked decrease of the intensity. In all samples, the width of the distribution is larger. After 72 h (Figure 3 lower panel), the values for the single-cell fluorescence intensity for the control sample are analogous to those observed after 24 h, whereas for all the samples treated with Cu(II), there is a dose-dependent decrease in the fluorescence intensity. Between 20 and 30 μg/mL, the median fluorescence intensity is almost half of that of the control, and at 100 and 500 μg/mL, there is almost no fluorescence emission at all.

Batch 2:

As for Batch 1, the fluorescence intensity is monitored for each single cell and plotted in a histogram. These histograms are provided in the Supplementary Materials. The range of concentrations of Cu(II) was restricted to values where a significant change in fluorescence intensity was observed for samples of Batch 1: i.e., at 10, 20, 30, and 100 μg/mL. Again, the fluorescence intensity of the samples was evaluated at time 0, 24, and 72 h.

**Figure 4:**
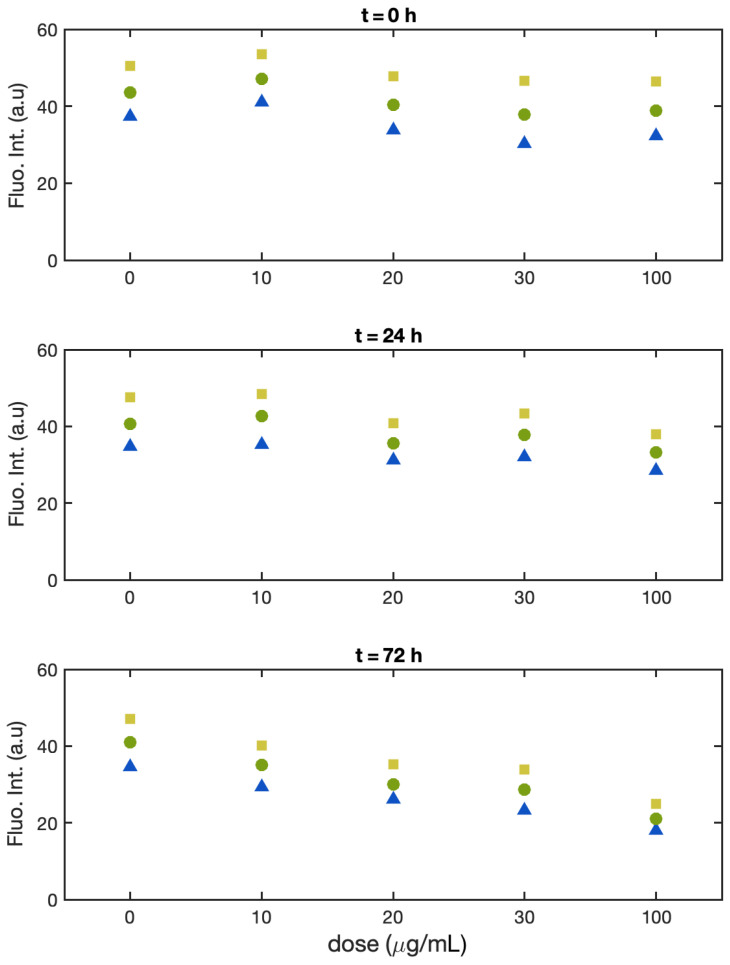
Batch 2: Normalized fluorescence intensity of the single alga cells at the 25th percentile (yellow square), median (green dot), and 75th percentile (blue triangle) as a function of Cu(II) concentration at t = 0 (**upper panel**), t = 24 h (**middle panel**), and t = 72 h (**lower panel**).

Figure 4 shows the intensities of the 25th percentile, median, and 75th percentile of the single cell fluorescence intensity distribution.

At time zero (Figure 4, upper panel), the fluorescence intensity falls in the same range for all solutions, and the width of the distribution is also similar with or without increasing doses of Cu(II). After 24 h (Figure 4, middle panel), all samples, including the control, show just a slight decrease in the fluorescence intensity (less than 5%), except for the one treated with the highest dose (100 μg/mL), which shows a 15% decrease. The width of the distribution is comparable for all sample. After 72 h (Figure 4, lower panel) the values for the single cell fluorescence intensity for the control sample are similar to those observed after 24 h, and for all the samples treated with Cu(II), we observed a dose-dependent decrease. At 20 and 30 μg/mL, the median fluorescence intensity showed a decrease of just 30% with respect to the control, and only at 100 μg/mL, the median of the fluorescence intensity reached half the value of the control sample. In this batch, the width of the distributions did not change significantly neither with the Cu dose nor with the time.

Batch 3:

As for Batches 1 and 2, the fluorescence intensity is monitored for each single cell and plotted in a histogram (for these data, see the Supplementary Materials). The range of concentrations of Cu(II) tested was 30, 100, 300, 500, and 700 μg/mL. The samples fluorescence intensity was evaluated at time 0, 24, and 72 h.

**Figure 5:**
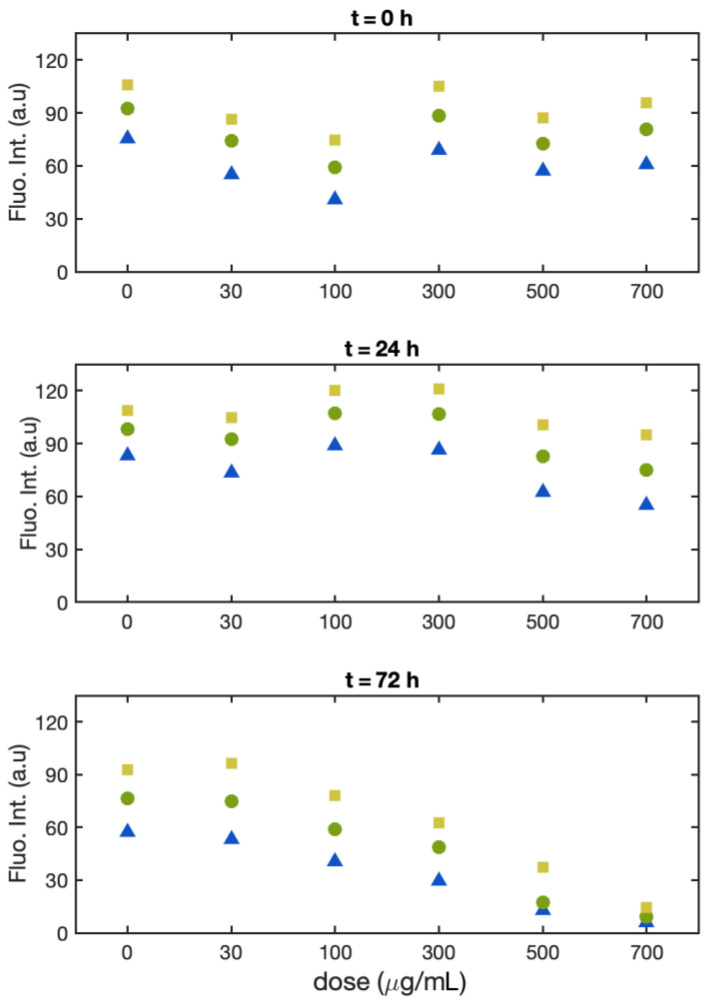
Batch 3: Normalized fluorescence intensity of the single alga cells at the 25th percentile (black square), median (red dot), and 75th percentile (blue triangle) as a function of Cu(II) concentration at t = 0 (**upper panel**), t = 24 h (**middle panel**), and t = 72 h (**lower panel**).

At time zero (Figure 5, upper panel), the fluorescence intensity falls in the same range for all solutions, except for 30 and 100 μg/mL, displaying a significantly lower value (20% and 30%, respectively). The width of the distribution was similar for all samples. After 24 h (Figure 5, middle panel), all the samples with Cu show fluorescence intensity similar to that of the control. In particular, the intensity for the samples with 30 and 100 μg/mL was higher than at time 0 and returned in line with the control sample. We have no experimental evidence of the origin of such behavior. We tentatively attribute it to a stabilization of the two cultures after they had been transferred or to possible contamination of the glass cells used to record the fluorescence intensity images at time 0. For all the concentrations and at all the times, the width of the distribution was comparable. After 72 h (Figure 4, lower panel), the single-cell fluorescence intensity for the control sample was lower with respect to 24 h (for the median 18%). In comparison with the control, at 100 and 300 μg/mL, the median fluorescence intensity showed a decrease of 27% and 40%, respectively. In this batch, the distributions width did not change significantly, either with the Cu dose or with the time.

Changes in the fluorescence intensity of chlorophyll *a* are a direct monitor of the well-being of plant cells. When these cells are exposed to external stress, there can be either an increase or decrease in fluorescence intensity which depends on the type of microalga investigated, the nature of the toxic agent, and the environmental conditions (type of medium, presence/absence of oxygen, pH etc.). In the case of Cu(II), there can be an increase in fluorescence intensity at low doses (order of tens of ng/mL), whereas at higher doses, it decreases because Cu(II) substitutes Mg(II) in chlorophyll *a* and turns off the fluorescence [37]. The decrease in fluorescence intensity is then associated with a total impairment of the photosynthetic cycle by Cu(II), and it signals complete disruption of the normal functions.

It is also known that microalgae show a marked decrease of chlorophyll *a* fluorescence intensity when they are heated up to temperatures of 80–100 °C. At these temperatures, the cells are dead and drastic changes in fluorescence intensity should be also related to cell death [38,39].

The anion Cl^¯^ can play a role in the oxidation of chlorophyll *a* and, therefore, in a decrease in the fluorescence signal [40].

In the following section, we discuss the changes in the values of median fluorescence intensity as a function of time and Cu(II) dose and as an index of highly stressed cells or even dead cells.

Since in Batch 3, we observed an oscillation in the median fluorescence up to 30%, we comment on changes of the median fluorescence intensity which are equal to or larger than 50%.

After 24 h, only Batch 1 showed a decrease in the median intensity of 50% at 100 μg/mL dose, whereas Batches 2 and 3 did not show significant change in median intensity at all the doses explored. After 72 h, there was a 50% decrease at approximately 20–30 μg/mL for Batch 1, at approximately 100 μg/mL for Batch 2, and at approximately 300 μg/mL for Batch 3. From these data, it is clear that cell culturing conditions can have a strong influence on the total impairment of the photosynthetic cycle as well as on cell viability, as signaled by the fluorescence decrease. As described in the Materials and Methods section, in Batch 1, only 0.5 mL of solution was sealed in a glass cell, whereas in Batches 2 and 3, 10 mL of solution was present. In Batch 1, the sample could not exchange O_2_ and CO_2_ with the environment, whereas gas exchange for Batches 2 and 3 were still possible since the systems were not gastight. Finally, in Batch 2 and Batch 3, the solutions were continuously stirred by the circular flow in the MF device or by the orbital shaker, respectively. The conditions used for cell culture in Batch 3 were the standard ones used to study microalgae cells and can be considered as the reference for other culture conditions. From our study, it appears clear that the use of MF devices with a large enough reservoir of sample (Batch 2) offers a culturing system that produces results comparable to (same order of magnitude) those observed in the usual culture conditions. The advantage, with respect to traditional cultures, lies in the possibility to directly examine the sample by simply disconnecting the MF chip from the pump and the reservoir, placing it under the microscope, and then reconnecting it for future examinations. This procedure avoids transferring cell solution from the reservoir (in our case, a falcon) to multiwells or single cells for inspection under the microscope, leading to accidental contamination of the sample.

The use of a small amount of sample for a prolonged time in an airtight system (Batch 1) appeared to accelerate the process of impairment of the photosynthetic cycle. There have already been studies showing that changes of the culturing conditions can modify the Cu(II) dose and time response of algae: Lombardi and Maldonado used batch and semi-continuous cultures to study the effect of Cu(II) on the growth rate and photosynthetic activity of *Phaeocystis cordata.* Batch cultures solution were maintained in the same medium for 5 days, whereas in semi-continuous cultures, a definite amount of medium was replaced daily to keep the population density constant. The growth rates for control samples where higher for semi-continuous cultures. The maximum quantum yield, measured with PAM fluorometry, was optimal and constant in the semi-continuous cultures, whereas it decreased significantly with culture age (0–72 h) in batch cultures [6]

The results obtained for Batch 1 were similar to those observed for *Chlorella vulgaris* exposed to increasing doses of CuCl_2_ grown inside 500 nl droplets generated in a segmented flow microfluidic device or in microtiter plates. In this work, a 50% decrease in cell autofluorescence was observed at Cu(II) concentrations of 35 μg/mL after 72 h exposure. As for Batch 1, the amount of sample, whereby the cells were cultivated, is extremely small, and the accumulation of waste produced by the dead cells can be detrimental [33].

Most of the works examining Cu(II) toxicity for freshwater microalgae monitor growth inhibition, enzyme activity, and the change in photosynthetic efficiency through PAM (pulse amplitude modulation) experiments of chlorophyll *a* fluorescence. These parameters reflect the stress induced by Cu(II) in microalgae cells which are still alive, and for this reason, they are characterized by EC_50_ responses to Cu(II) concentrations which are from one to three orders of magnitude lower than the one observed in this work; therefore, the comparison with these experiments is not straightforward [5,7,41]

### 3.2. Single-Cell FLIM Experiments

**Figure 6:**
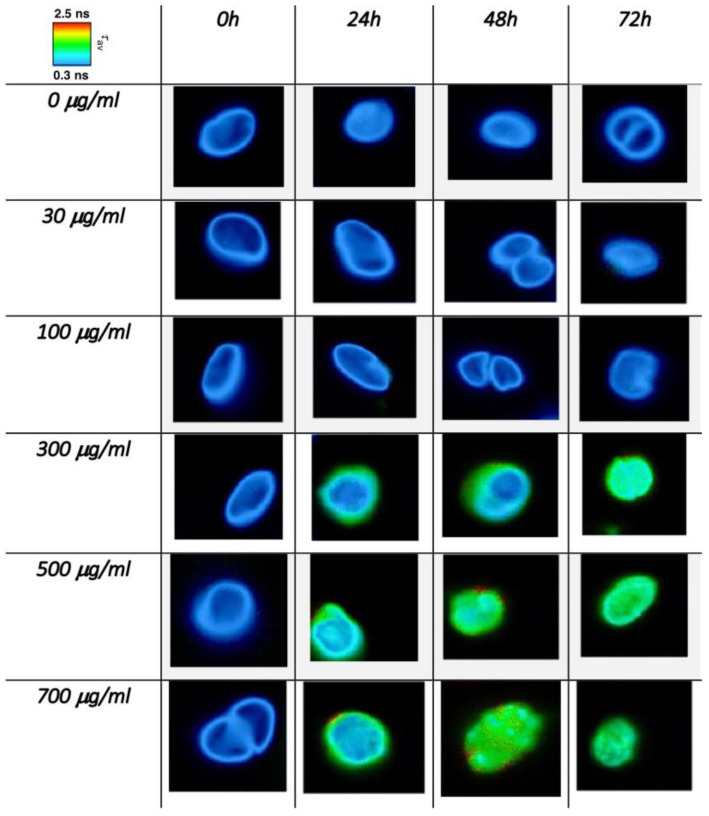
FLIM images of *C. cimbrica* cell as a function of dose and time. The color scale maps the intensity mediated average fluorescence lifetime (see text for further explanations).

FLIM experiments are carried out only with samples of Batch 3. These experiments allow observation of the change in the decay dynamic of chlorophyll *a* at increasing concentrations of Cu(II) and as a function of time. These experiments record the fluorescence decay signal along different positions in the sample, generating a 2D image of the sample on the basis of the parameters extrapolated from a fit of the decays. A three-term multi-exponential function fits these decays well. The first term has a time constant of the order of 100 ps or less and is shorter than the characteristic time resolution of our set-up. It can be attributed to not only very fast decay processes involving chlorophyll *a*, but it can also come from laser-scattered light, which is not properly rejected by the filter. In the data analysis of all samples, this component was fixed to 100 ps and is not further discussed. The second and third terms, instead, were free to change and had characteristic decay times in the ranges of 200 ÷ 500 ps (*τ*_1_) and 1000 ÷ 2300 ps (*τ*_2_), with associated amplitudes *A*_1_ and *A*_2_, respectively. Two-dimensional FLIM maps can be constructed from these data. In Figure 6, maps of *C. cimbrica* cells displaying the intensity-weighted average fluorescence lifetime:

$$\tau_{Av}=\frac{\sum_{i}A_{i}\tau_{i}^{2}}{\sum_{i}A_{i}\tau_{i}}$$

are shown. Since the intensity *A_i_τ_i_* is proportional to the number of photons emitted by fluorophores having lifetime *τ_i_*, then the average lifetime *τ_Av_* is weighted with respect to the contribution of each type of fluorophore to the total emission.

**Figure 7:**
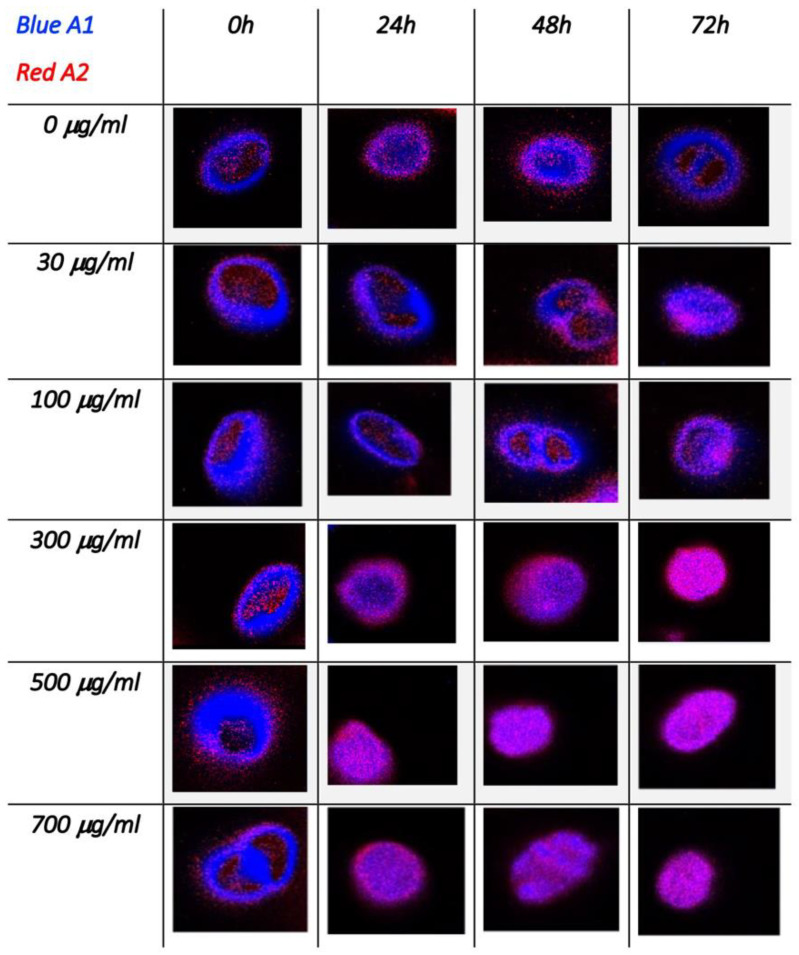
FLIM images of *C. cimbrica* cell as a function of dose and time. These maps show the amplitudes of the two exponential decay component: the shorter one (300–500 ps) in blue and the longer one (1.2–2.2 ns) in red (see text for further explanations).

Figure 6 shows these maps for single *C. cimbrica* cells as well as some cell clusters. These cells were chosen among the 10 measured for each time and dose because they are representative of most of the analyzed cells. On the upper left corner of Figure 6, there is a color code map for the different *τ_Av_*: blue indicates the lower time of 0.3 ns, whereas red is the longest time of 2.5 ns. Along the column, Cu(II) dose increases, whereas along the row, time increases. At time zero for all the doses explored, the average lifetime is very fast (~300 ps); after 24 h, for doses equal or higher than 300 μg/mL, there is a lengthening of the average lifetime, in particular along the cell borders, where the average lifetime is approximately 1 ns. After 48 and 72 h at the higher doses of 500 and 700 μg/mL, the average lifetime is of the order of 1 ns in the entire cell. Figure 7 shows the same *C. cimbrica* cells depicted in Figure 6, but instead of the average time, the amplitude *A*_1_ (blue) and *A*_2_ (red) associated with the two characteristic decay times are reported. At zero time and low doses, the blue component, associated with the shorter lifetime, dominates. After 24 h, there is a marked increase of the red component for doses equal or higher than 300 μg/mL, and this component become even more important after 48 and 72 h, again for doses equal to or higher than 300 μg/mL.

**Figure 8:**
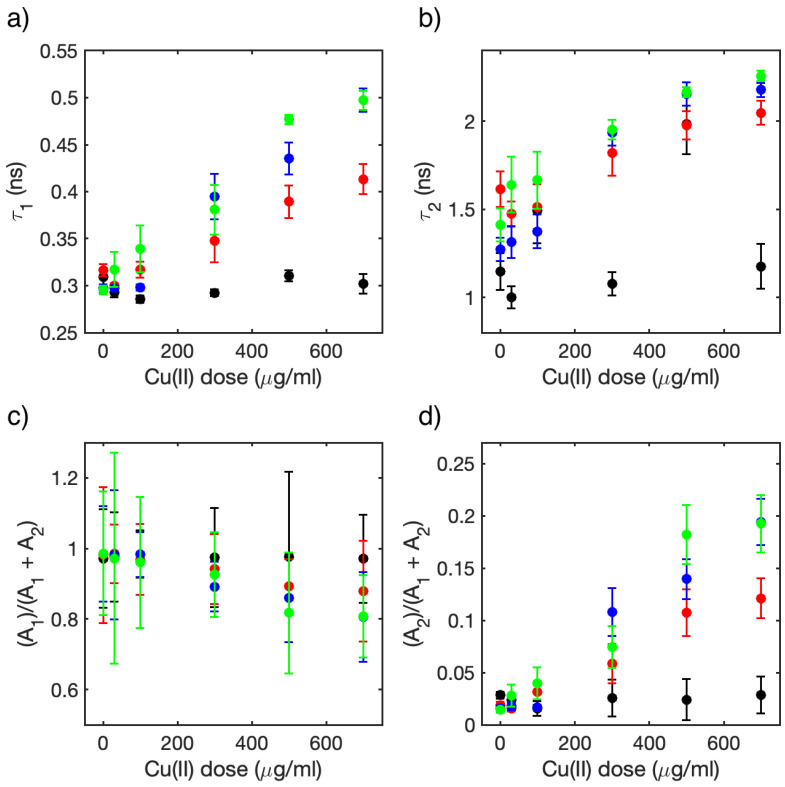
Average values of the parameters for the bi-exponential functions used to fit the 10 FLIM images of the *C. cimbrica* cells for each dose and time; in all plots black represents t = 0 h, red t = 24 h, blue t = 48 h, and green t = 72 h: (**a**) short time constant, (**b**) long time constant, (**c**) relative value of the area of the decaying signal associated to the short component (see text), and (**d**) relative value of the area of the decaying signal associated long component (see text).

Figure 8 shows the parameters retrieved from the exponential fit. Figure 8a,b shows the average decay time constant for the short and long decay, respectively. These average data have been calculated considering all 10 FLIM images of the cells at a specific dose and time. Error bars were calculated from standard deviation. Both decay time constants showed an increase with both Cu (II) dose and time, and they almost doubled after 72 h and at the highest concentration: 500 and 700 μg/mL. Figure 8c,d depicts the fractional fluorescence intensity, i.e., the relative amplitude for the two components: *A_i_*/(*A*_1_+*A*_2_) where *i* is 1 and 2, respectively. This parameter describes the ratio of the excited chlorophylls which decay with the specific time constant. The short component dominates (value of 0.95) at time zero for all doses and at 48 and 72 h for doses up to 100 μg/mL. It decreased by almost 20% after 72 h for doses higher than 300 μg/mL. The long component was negligible at time zero for all doses, instead it reached almost 20% at 48 and 72 h and for doses higher than 300 μg/mL. The error bars for the *A*_1_/(*A*_1_+*A*_2_) ratio exceeded 1. Although this is not physically correct, it still marks the relative uncertainty affecting our data when error bars are calculated using the error propagation formula.

Chlorophyll *a* fluorescence lifetime in isolated photosystems as well as in plant leaves is often characterized by a multi-exponential decay. The fastest component, shorter than 100 ps, is usually attributed to chlorophyll *a* emission from PSI [42]. It is difficult to observe this component because the PSI fluorescence quantum yield is very small. Two longer components with decay time constants in the hundreds of ps (200–500 ps) and nanoseconds (1.0–1.7 ns) come from emission of chlorophyll *a* in PSII. These two different kinetic processes are attributed to the “open” and “closed” condition for PSII. In the first case, the photosynthetic cycle is active, whereas in the second case, it is inactive. Fluorescence decay time is shorter in the first process because energy transfer to the reaction center is active, whereas in the second case, it is longer since this process is not active [13]. Few FLIM experiments have been carried out on microalgae. S. Nozue and coworkers [12] investigated the effect of increasing laser power on the cyanobacterium *Anabaena variabilis* and on green alga *Parachlorella kessleri*. The authors observed that the average decay time calculated from FLIM maps increased as the laser power was increased for both systems. The fluorescence decays were well simulated by a bi-exponential function with characteristic times of 0.20–0.32 ns and 0.8–1.4 ns for *Parachlorella kessleri.* They attributed the short and long components to the “open” and “closed” state of PSII. The short component, identified with the ‘open’ state, dominated at low laser powers, whereas the longer component, attributed to the ‘closed’ state, gained importance at high laser powers.

Y. Wu et al. [43] explored the toxic effects of Hg(II) and methylmercury (MeHg) on *Thalassiosira weissflogii* through flow cytometry and FLIM. FLIM experiments showed an increase of the average lifetime of chlorophyll *a* fluorescence as the Hg(II) dose increased, whereas increasing doses of MeHg did not show any change in average fluorescence lifetime, indicating that MeHg had no influence on the photosynthetic cycle. Flow cytometry experiments showed growth inhibition for both Hg(II) and MeHg, although for Hg(II) the effect was due to an increase of injured cells, whereas for MeHg, reduced cell division was invoked.

Following these works, we attribute the short and long components to the emission of chlorophyll *a* in the “open” and “closed” conditions inside PSII, respectively. The lengthening of the short component can be interpreted as an impairment of the energy transfer processes from the excited chlorophylls to the reaction center prompted by Cu(II). A rough estimate of the decreases in efficiency of the energy transfer process can be gained from the average value of *τ*_1_ in the presence (w) and absence (w/o) of Cu(II). The Förster energy transfer model evaluates the energy transfer efficiency E from the donor (D) to the acceptor (A) as: E = 1 − *τ*_DA_/*τ*_D_, where *τ*_DA_ and *τ*_D_ are the lifetimes of the donor with and without the acceptor, respectively. This formula cannot be applied straightforwardly to our data because the system is much more complex than the simple donor–acceptor pair, but we still can consider the ratio of the lifetimes *τ*_1_(w)/*τ*_1_(w/o) as an estimate of the relative change in energy transfer efficiency. For example, at time 0, the average value of *τ*_1_ was almost equal at all doses, meaning that the Cu(II) took some time to influence the photosynthetic cycle. The value of *τ*_1_(w/o) did not change with time up to 72 h. After 72 h, the value of the ratio *τ*_1_(w)/*τ*_1_(w/o) increased from 1.06 for the 30 μg/mL Cu(II) dose to 1.3 and 1.6 for the 100 μg/mL and 700 μg/mL doses. This means that energy transfer and, therefore, photosynthetic efficiency had decreases of almost 30% and 50% for the two doses. In addition, the amplitude associated with the *τ*_1_ time (Figure 8c) had decreases of almost 20%, confirming the decrease of systems in the “open” condition.

The increase of the average time *τ*_2_, associated with the “closed” photosynthetic systems as time and dose increased, can be associated with a further change in the decay pathways of the excited chlorophyll *a*, which are already excluded from the photosynthetic cycle. Data in the literature show that at low concentrations in organic solvents, the lifetime of excited chlorophyll *a* is approximately 5–6 ns, and when water is added, aggregation occurs and the lifetime, observed through the decay of fluorescence, shortens considerably [44,45]. Following these studies, it is possible to attribute the increase in *τ*_2_ to a progressive disruption of the photosystems structure in which chlorophylls are aggregated, preventing self-quenching phenomena between nearby chlorophylls. The increases of the relative amplitude (Figure 8d) of this component confirms that the relative number of “closed” photosystems increases at high Cu(II) doses.

## 4. Conclusions

Experiments investigating the single-cell fluorescence of *Coccomyxa cimbrica* as a function of increasing doses of Cu(II) at 0, 24, and 72 h are presented. In these experiments, different in vitro culturing conditions are used: one comparable to microwells condition (Batch 1), the second under continuous flow in a microfluidic device (Batch 2), and the last one in plastic falcons continuously stirred by an orbital shaker (Batch 3). After 72 h, the median single cell fluorescence intensity decreased by almost 50% at doses of 20–30 μg/mL for Batch 1, whereas for Batches 2 and 3, this change occurs at doses of the order of 100–300 μg/mL. We attribute this behavior to two factors: the limited amount of solution and the absence of oxygen exchange for Batch 1 relative to the other two. These experiments show that growth in a microfluidic device, such as the one proposed, provides the same results as the one obtained using standard examinations protocols, such as those used in Batch 3. The advantage in using microfluidic devices compared with traditional culture condition lies in the ability to avoid accidental contamination of the sample since there is no need to withdraw sample from the batch to examine it at the different time intervals.

FLIM data confirm what is already known about Cu(II) toxicity towards photosynthetic systems. From the analysis of FLIM data, two characteristic decay time constants are attributed to the lifetime of chlorophyll *a* in PSII in the “open” (hundreds of picoseconds) and “closed” configuration (nanoseconds). There is a steady and marked increase of both these values in time as the dose of Cu(II) increases. The lifetime of the short component is associated with a decrease in efficiency of the energy transfer process towards the reaction center, whereas for the long component disruption of the aggregates structure is invoked.

## Data Availability

Not applicable.

## Supplementary Materials

The following supporting information can be downloaded at: https://www.mdpi.com/article/10.3390/bios13040417/s1, Figure S1: Sketch and picture of the microfluidic set up; Figure S2: Fluorescence decay curves measured with the FLIM technique in samples without and with Cu(II) after 72 h; Figure S3: Single-cell normalized fluorescence intensity distributions for all three batches at selected Cu(II) doses and at times 0, 24, 48, and 72 h.
