# Supplementary material for: Response of Coccomyxa cimbrica sp.nov. to Increasing Doses of Cu(II) as a Function of Time: Comparison between Exposure in a Microfluidic Device or with Standard Protocols
Source: Biosensors (Basel). 2023 Mar 23;13(4):417. doi: 10.3390/bios13040417 (PMC10135970; doi:10.3390/bios13040417)

Supplementary Material

- Figure S1: Sketch and picture of the microfluidic set up.
- Figure S2: Fluorescence decay curves measured with the FLIM technique in samples without and with Cu(II) after 72 h
- Figure S3: Single cell normalized fluorescence intensity distributions for all the 3 batches at selected Cu(II) dosed and at time 0, 24, 48, and 72 hours.

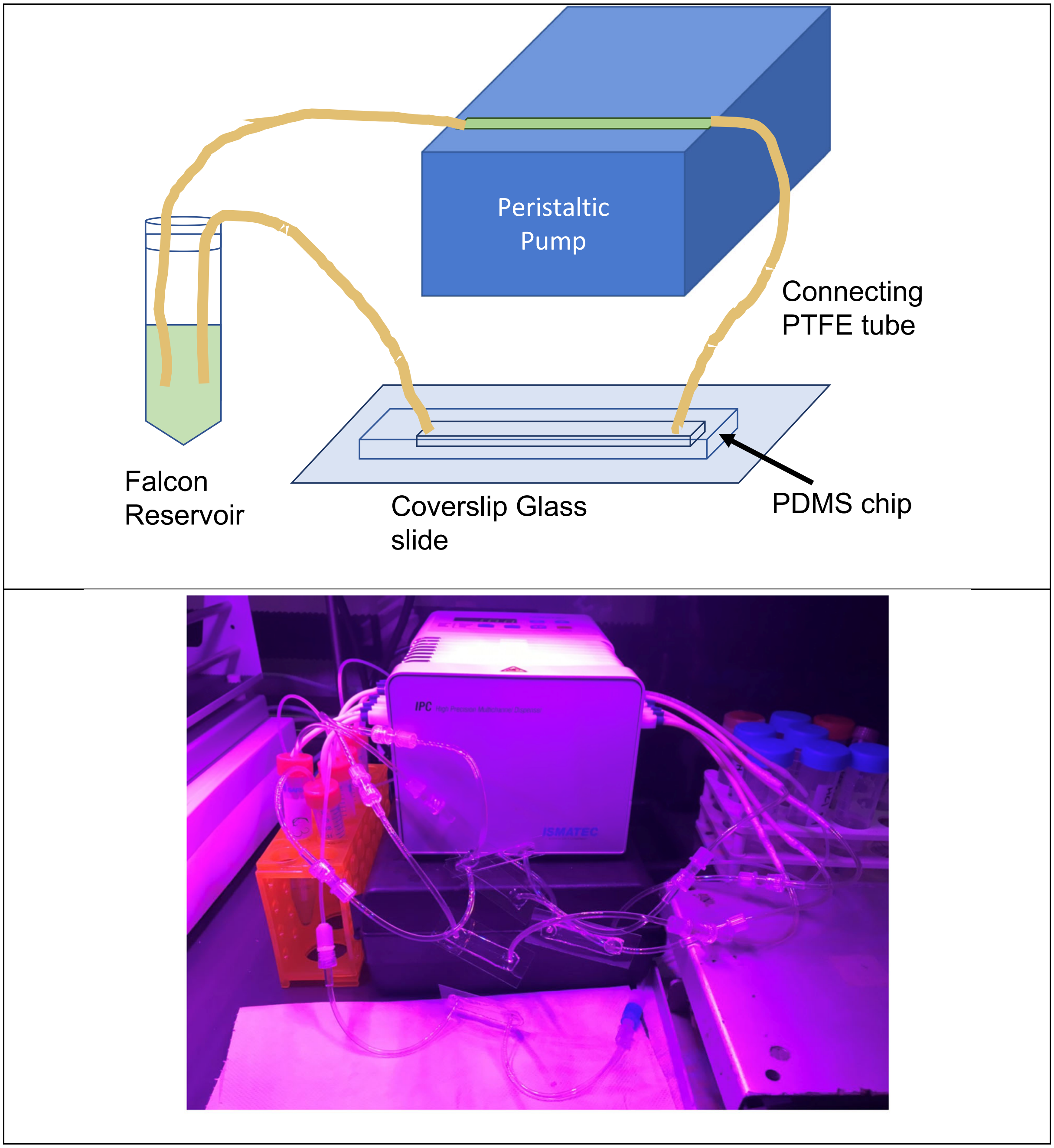

Figure S1: Upper panel: scheme of the microfluidic system set up for microalgae cultivated in batch 2. Lower panel a photograph of the set up, showing 5 microfluidic chips connected to the peristaltic pump.

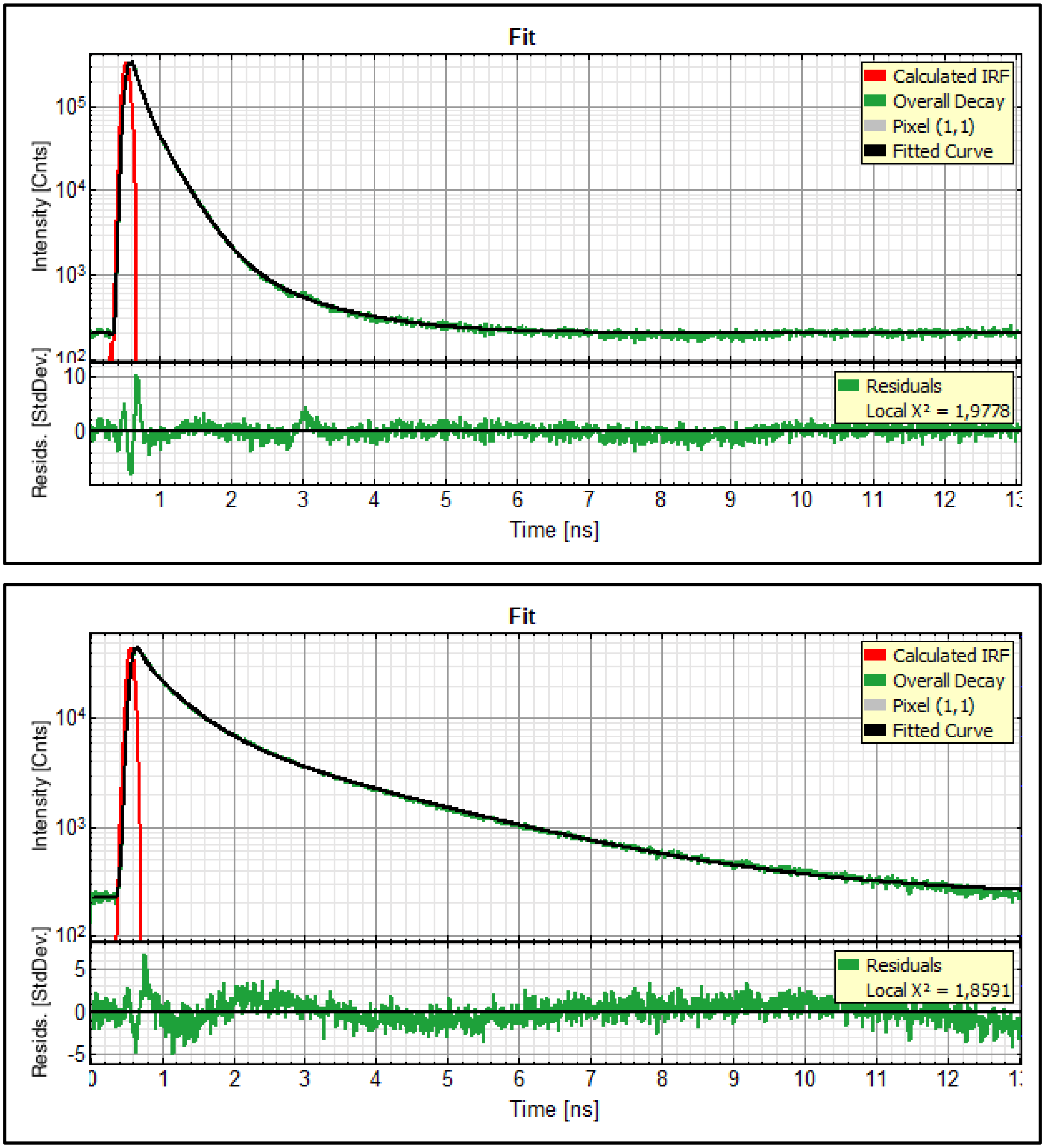

Figure S2: Fluorescence decay (green), IRF (red) and fitting curve (black) measured for *C.cimbrica* cells of batch 3 with 0 μg/ml (upper panel) and 700 μg/ml (lower panel) of Cu(II) after 72 h.

Figure S3: Single cell normalized fluorescence intensity distributions for all the 3 batches at selected Cu(II) dosed and at time 0, 24, 48, and 72 hours.

<u>Single cell fluorescence intensity distribution for Batch 1</u>

Cu(II) = 0 μg/ml

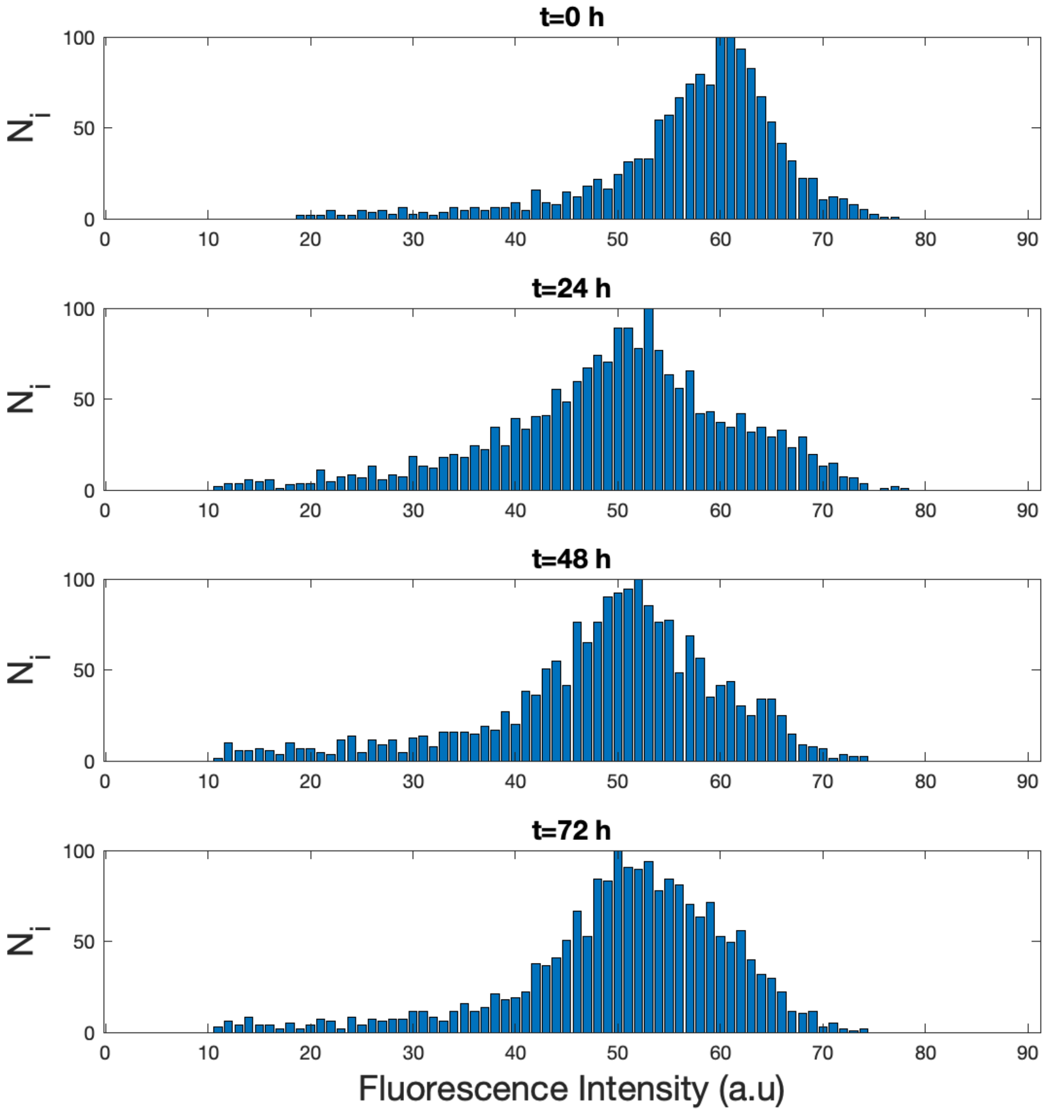

Single cell fluorescence intensity distribution for Batch 1

Cu(II) = 10 μg/ml

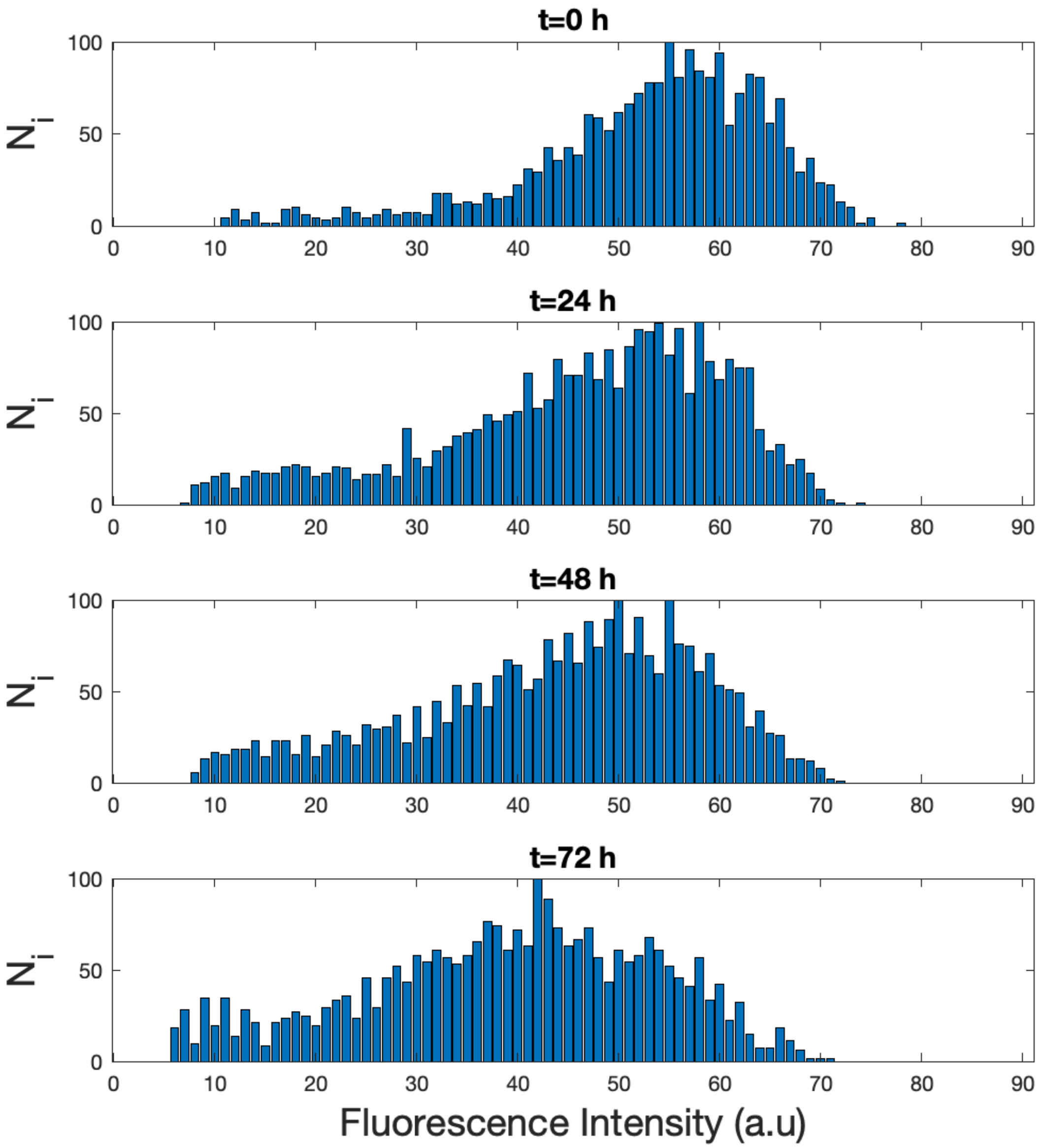

Single cell fluorescence intensity distribution for Batch 1

Cu(II) = 30 μg/ml

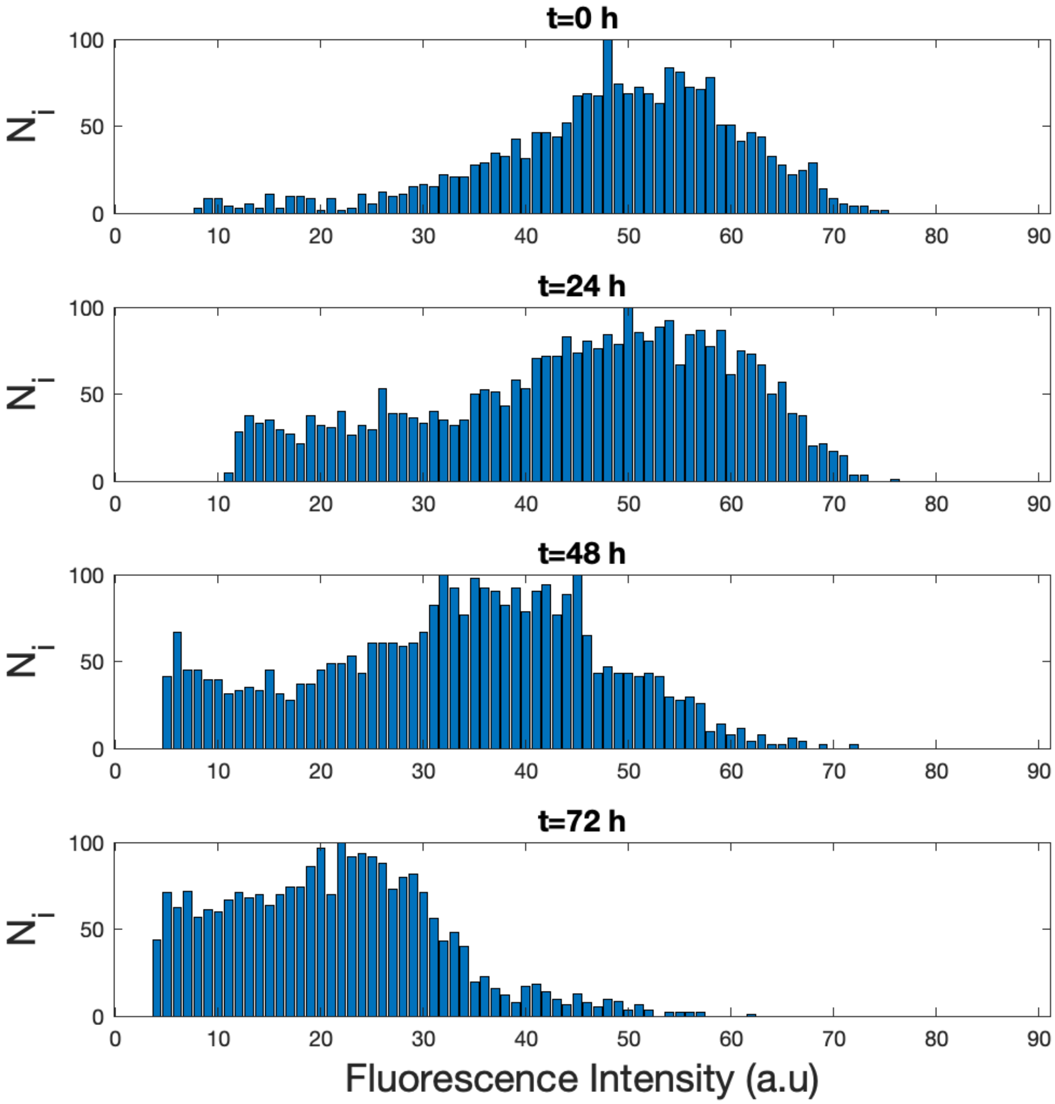

Single cell fluorescence intensity distribution for Batch 1

Cu(II) = 100 μg/ml

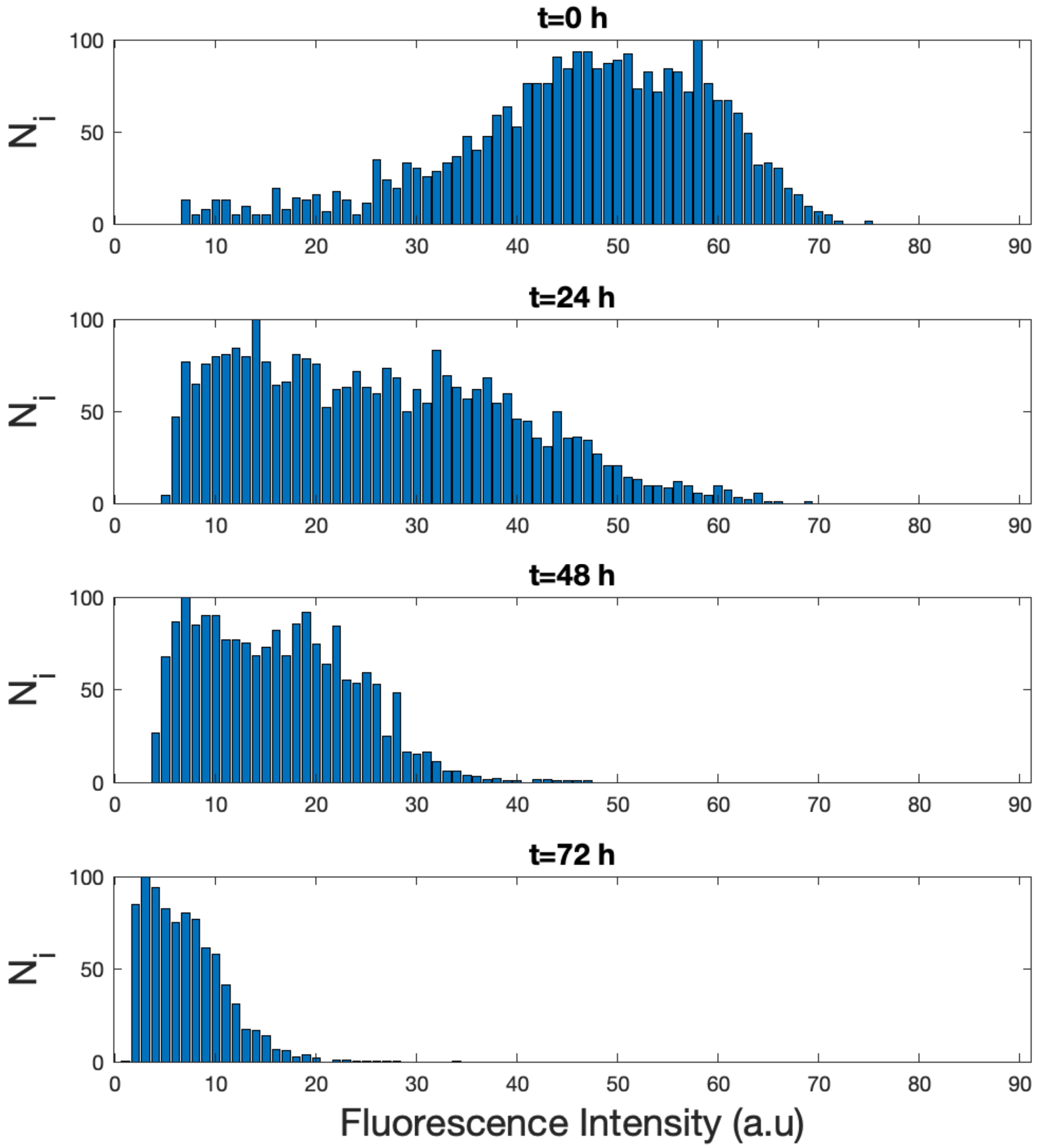

Single cell fluorescence intensity distribution for Batch 2

Cu(II) = 0 μg/ml

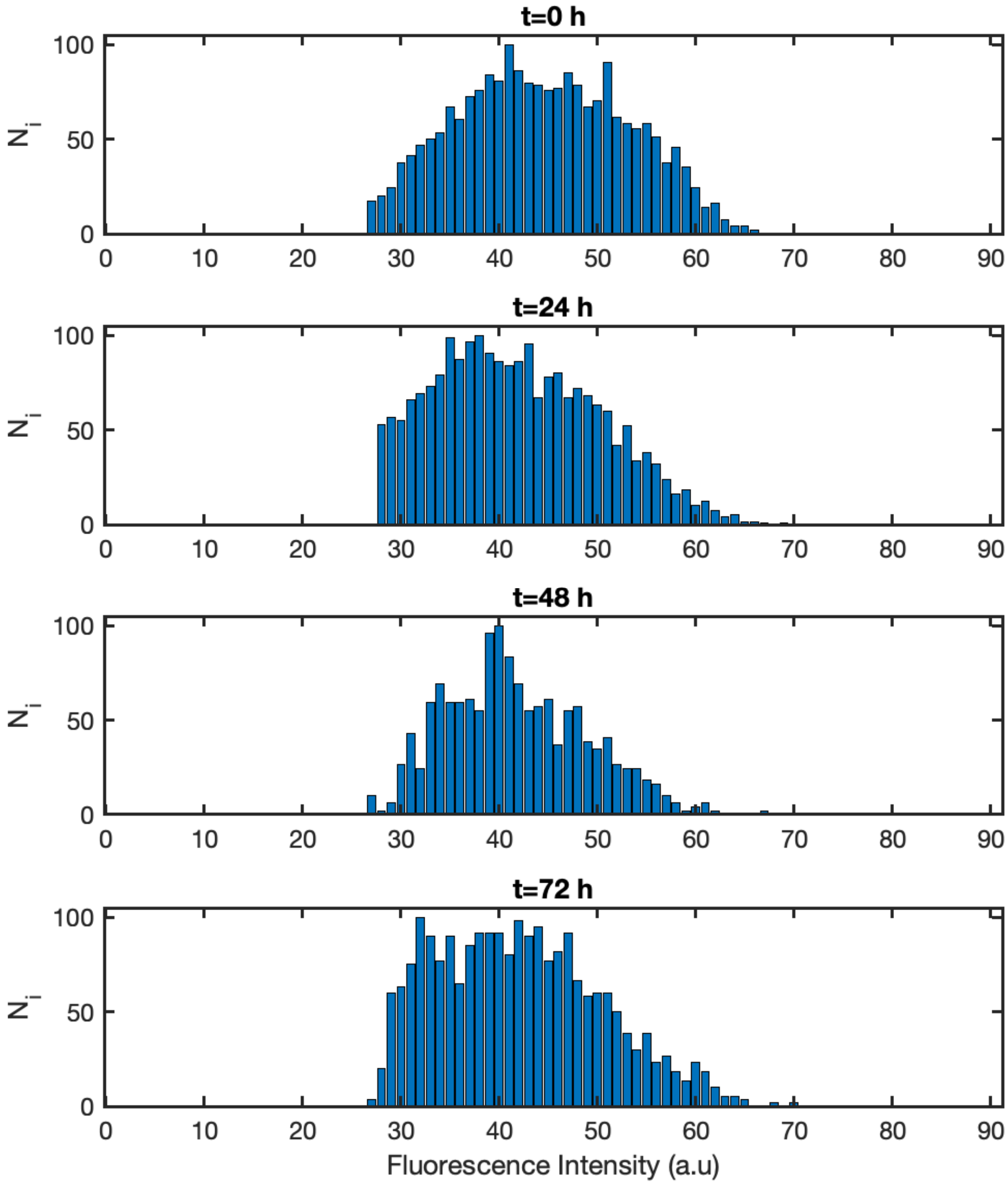

Single cell fluorescence intensity distribution for Batch 2

Cu(II) = 30 μg/ml

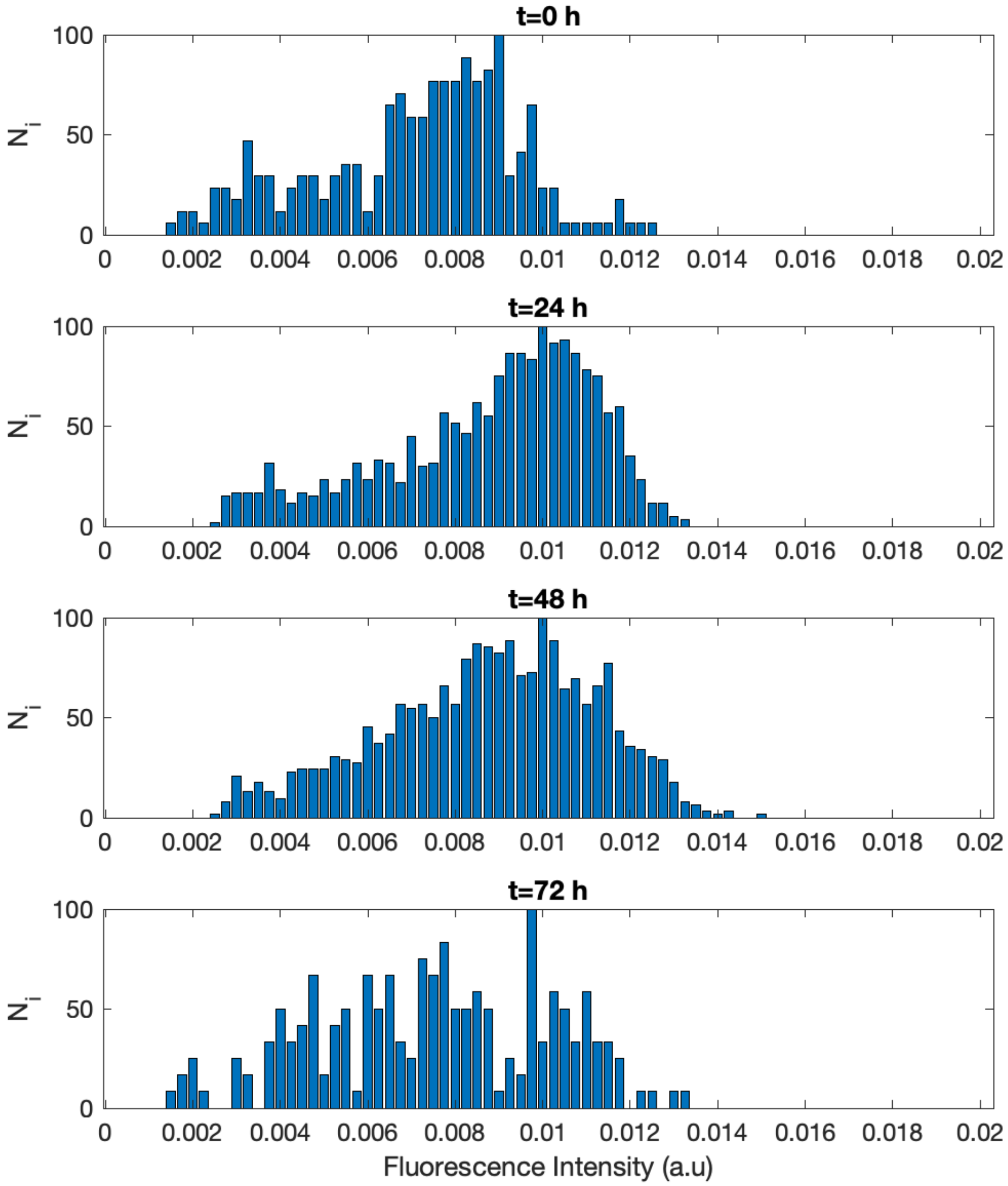

Single cell fluorescence intensity distribution for Batch 2

Cu(II) = 100 μg/ml

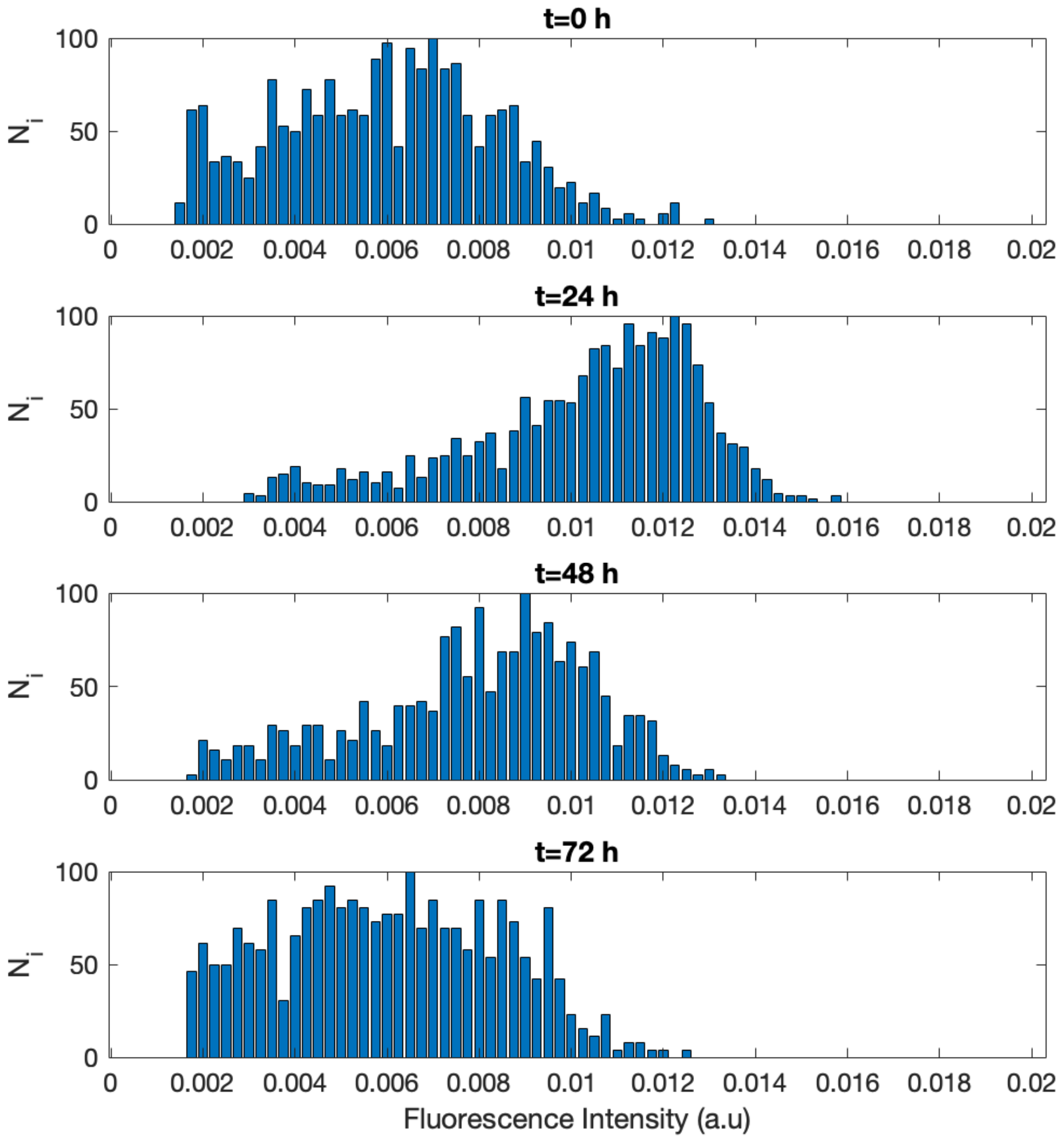

Single cell fluorescence intensity distribution for Batch 2

Cu(II) = 500 μg/ml

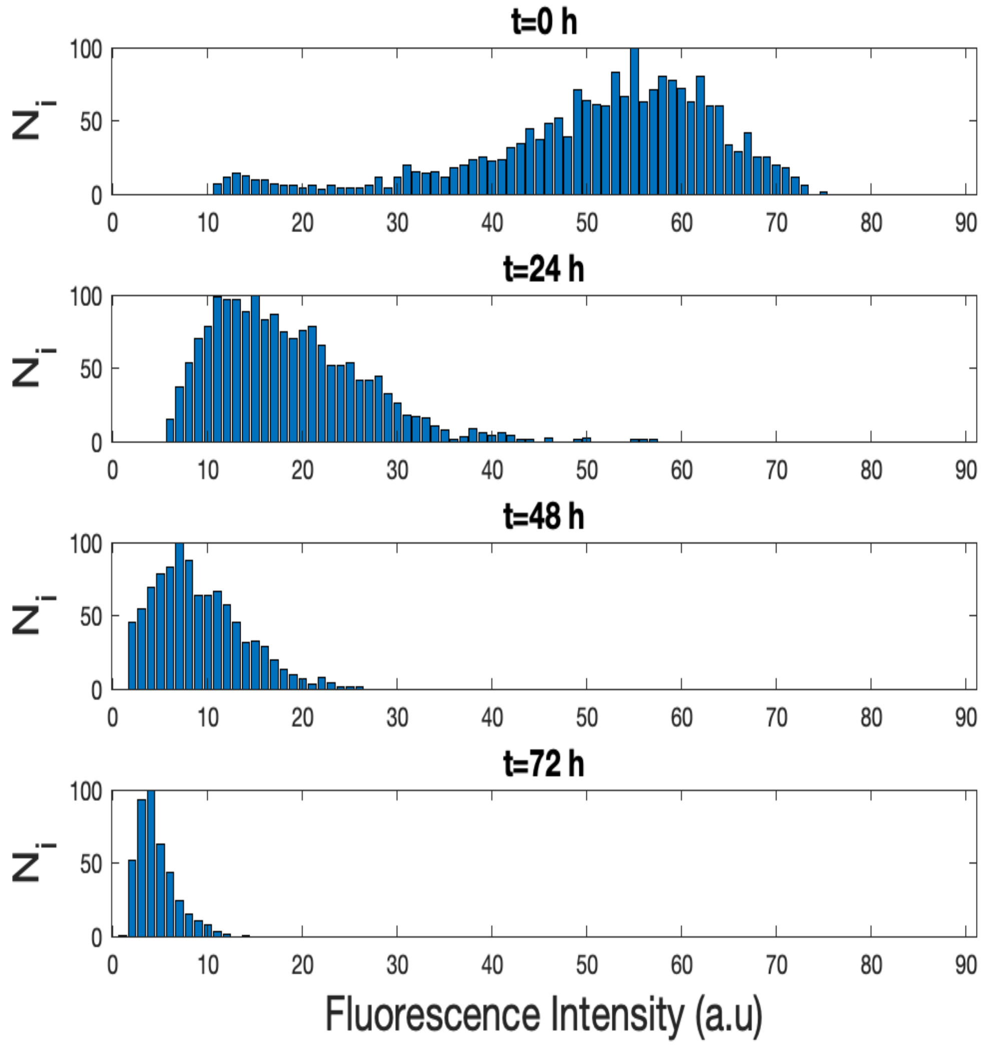

Single cell fluorescence intensity distribution for Batch 3

Cu(II) = 0 μg/ml

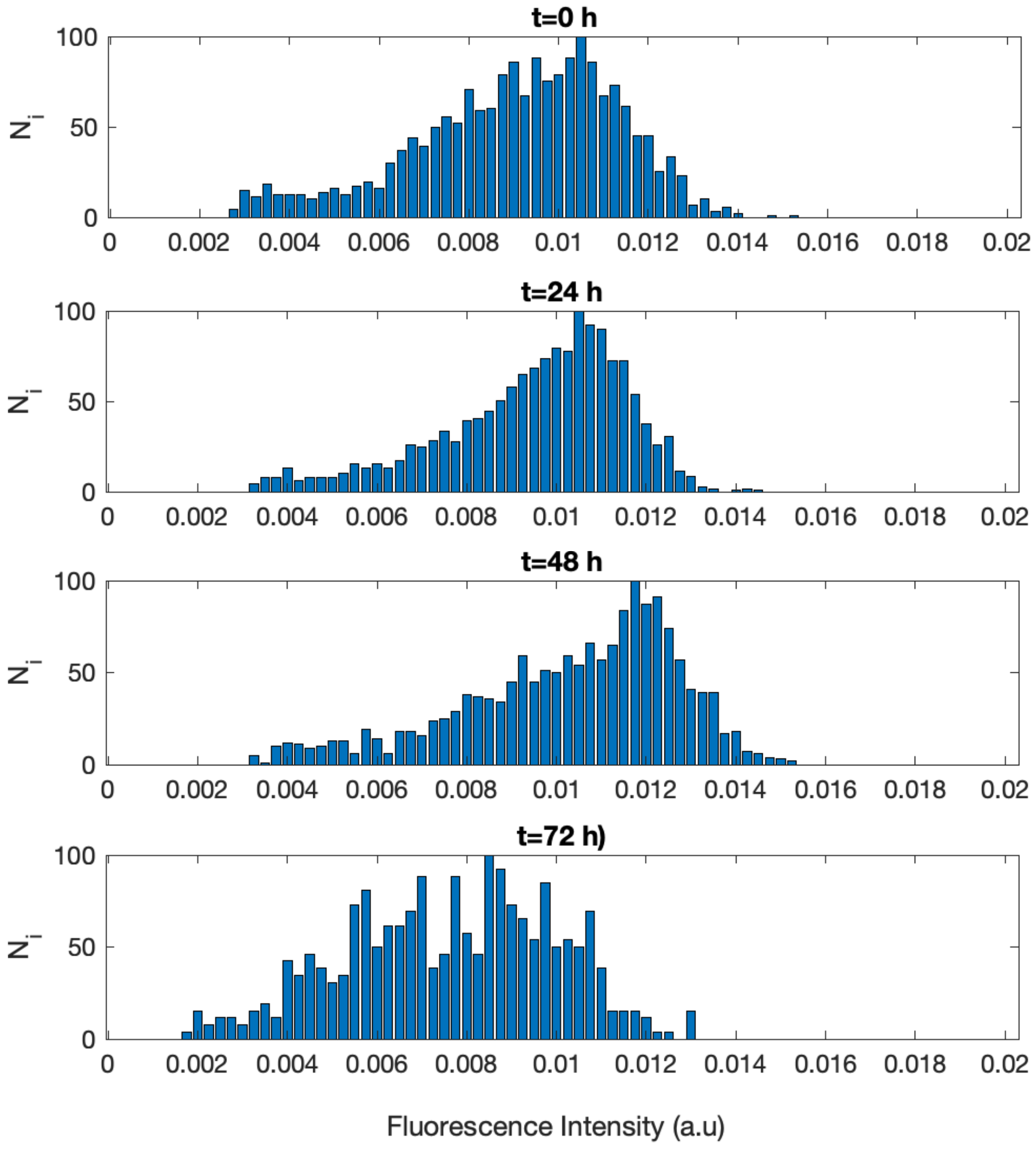

Single cell fluorescence intensity distribution for Batch 3

Cu(II) = 30 μg/ml

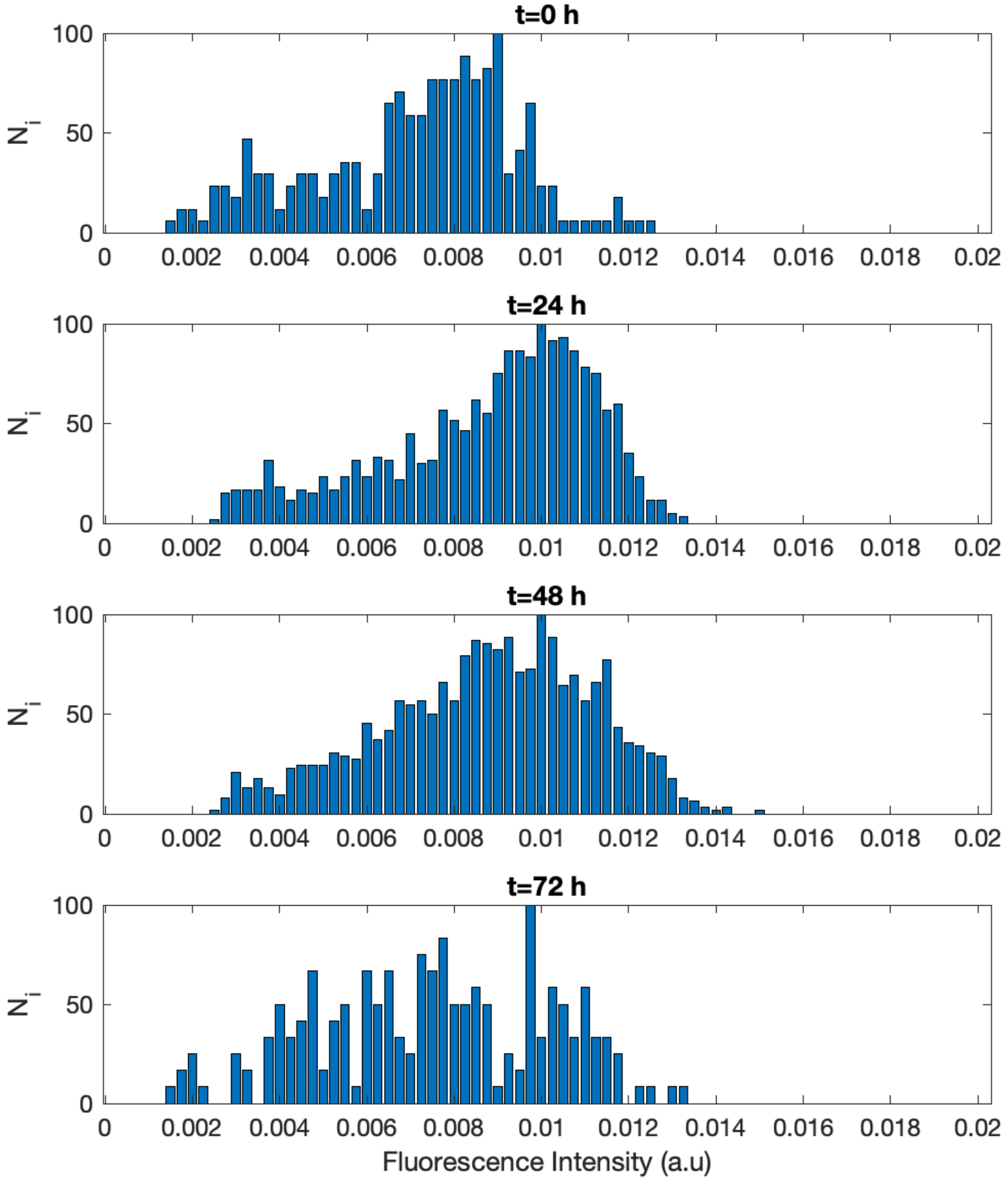

Single cell fluorescence intensity distribution for Batch 3

Cu(II) = 100 μg/ml

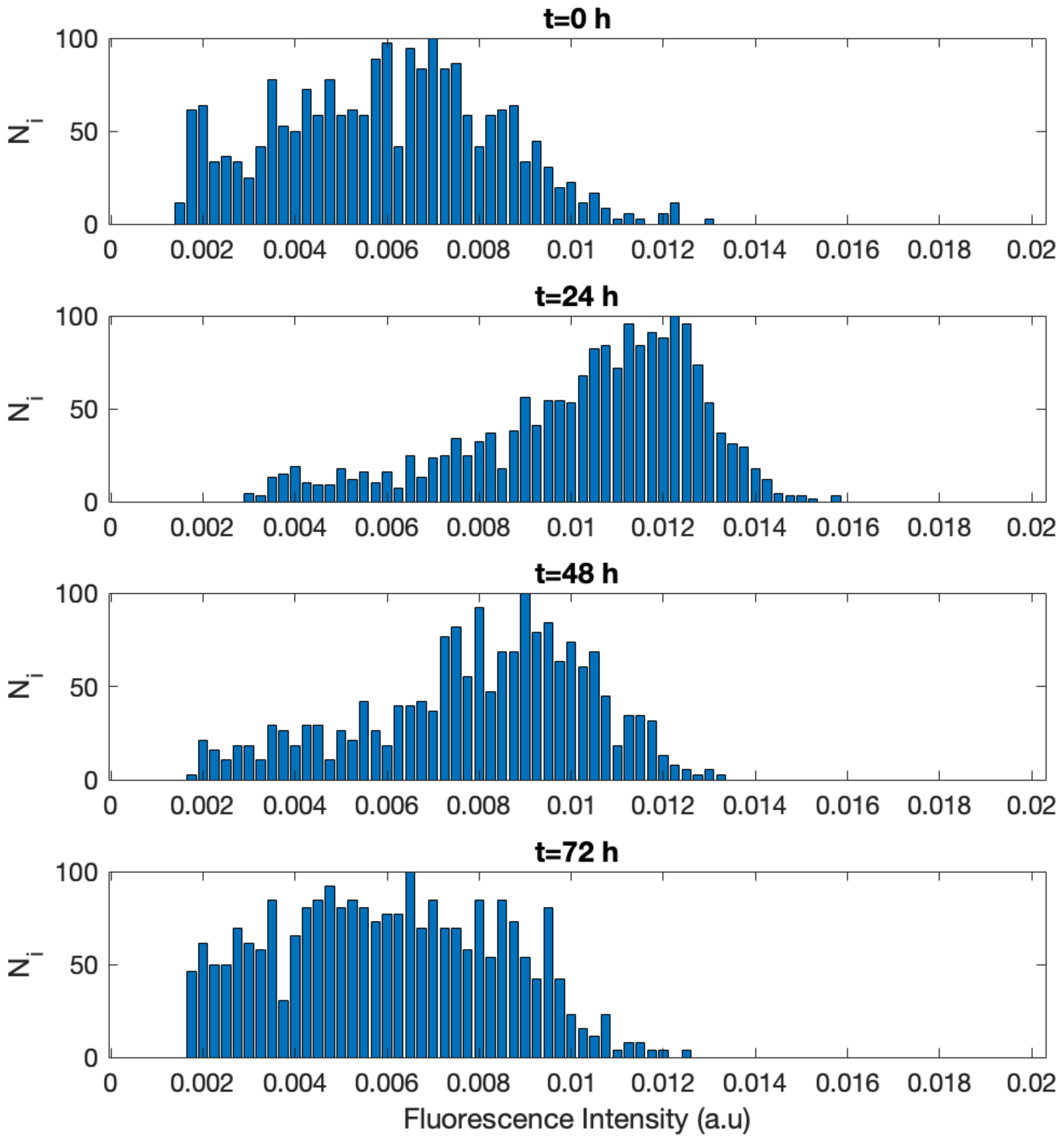

<u>Single cell fluorescence intensity distribution for Batch 3</u>

Cu(II) = 700 μg/ml

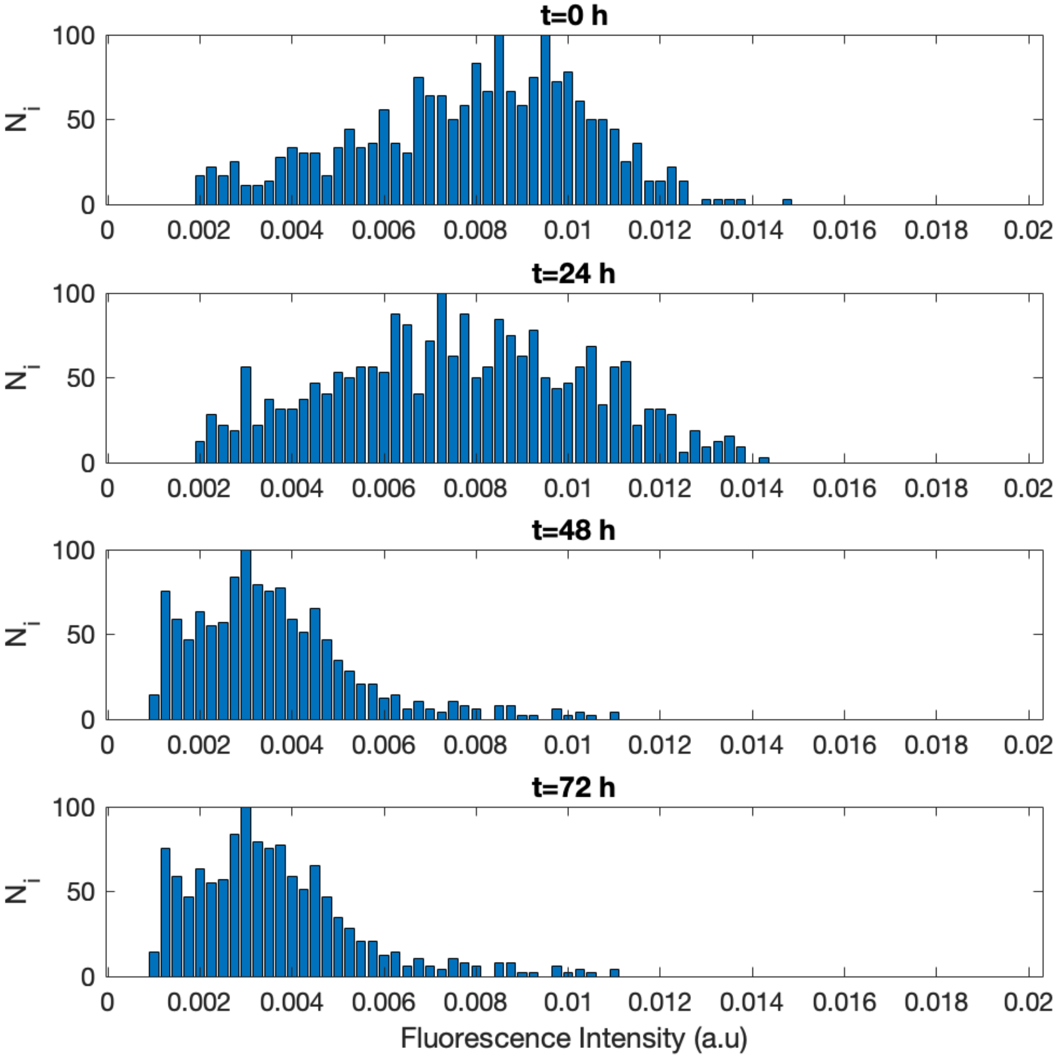

Supplement: Supplementary file 1 [file biosensors-13-00417-s001.zip › biosensors-2273505-supplementary.pdf]
